# Roles of Reactive Oxygen Species and Mitochondria in Seed Germination

**DOI:** 10.3389/fpls.2021.781734

**Published:** 2021-12-09

**Authors:** Muhammad Awais Farooq, Xiaomeng Zhang, Muhammad Mubashar Zafar, Wei Ma, Jianjun Zhao

**Affiliations:** ^1^State Key Laboratory of North China Crop Improvement and Regulation, Key Laboratory of Vegetable Germplasm Innovation and Utilization of Hebei, Collaborative Innovation Center of Vegetable Industry in Hebei, College of Horticulture, Hebei Agricultural University, Baoding, China; ^2^Department of Plant Breeding and Genetics, University of Agriculture, Faisalabad, Pakistan; ^3^Institute of Cotton Research, Chinese Academy of Agricultural Sciences, Anyang, China

**Keywords:** seed germination and dormancy, reactive oxygen species (ROS), mitochondria, heat shock proteins (HSPs), embryogenesis and endosperm

## Abstract

Seed germination is crucial for the life cycle of plants and maximum crop production. This critical developmental step is regulated by diverse endogenous [hormones, reactive oxygen species (ROS)] and exogenous (light, temperature) factors. Reactive oxygen species promote the release of seed dormancy by biomolecules oxidation, testa weakening and endosperm decay. Reactive oxygen species modulate metabolic and hormone signaling pathways that induce and maintain seed dormancy and germination. Endosperm provides nutrients and senses environmental signals to regulate the growth of the embryo by secreting timely signals. The growing energy demand of the developing embryo and endosperm is fulfilled by functional mitochondria. Mitochondrial matrix-localized heat shock protein GhHSP24.7 controls seed germination in a temperature-dependent manner. In this review, we summarize comprehensive view of biochemical and molecular mechanisms, which coordinately control seed germination. We also discuss that the accurate and optimized coordination of ROS, mitochondria, heat shock proteins is required to permit testa rupture and subsequent germination.

## Highlights

- ROS inside cell due to external environmental dynamics signals seed germination.
- Mitochondria produce large amounts of ATP for seed germination.
- HSPs induces testa weakening and micropylar endosperm decay to release dormancy.

## Introduction

**FIGURE 1:**
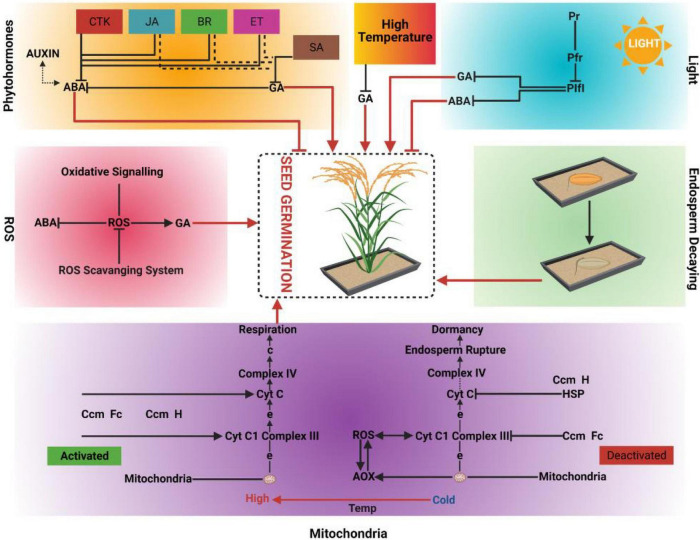
Driving forces of seed germination; Phytohormones, High temperature, Light, Reactive oxygen species (ROS), Endosperm decaying and Mitochondria. CTK: Cytokinins, JA: Jasmonic acid, BR: Brassinosteroids, ET: Ethylene, SA: Salicylic acid, GA: Gibberellic acid, ABA: Abscisic acid, PIF1: Phytochrome Integrating Factor 1, Pfr: Photoreceptors far red, Pr: Photoreceptor red, Cyt c: Cytochrome c, AOX: Alternative oxidases, HSP: Heat shock proteins, ROS: Reactive oxygen species, Temp: Temperature.

Seed germination is crucial for uniform and maximum crop production (Carrera-Castaño et al., 2020), and the optimization of intrinsic and extrinsic factors is very important for promoting the transition from dormancy to germination (Figure 1) (Wang et al., 2020). The dormancy level of commercial crop varieties is typically lower than that of their wild relatives, as this helps to achieve maximum production. Nonetheless, the level of dormancy must be calibrated to prevent pre-harvest sprouting and consequent losses of yield and quality (Shu et al., 2016). A deeper understanding of seed germination and dormancy is therefore important for both agronomic and economic reasons.

The transition from seed dormancy to germination is a physiological process that is regulated by diverse endogenous hormones and environmental factors. ABA (abscisic acid) and GAs (gibberellins) are the main hormones that work in coordination to release seed dormancy and enable seed germination (Wang et al., 2019). Recent findings have revealed that auxins also play an important role in maintaining seed dormancy until conditions are favorable for plant survival (Shuai et al., 2017). Besides intrinsic cues, numerous extrinsic factors can prolong or terminate seed dormancy and promote seed germination and development. Light (Oh et al., 2004), temperature (He et al., 2016), and soil conditions (Meng et al., 2017) are major signals that can be perceived by seeds to regulate the timing of germination. The regulatory effect of light on seed germination depends on its spectrum, the red light increases the seed germination than any other light color (Stawska and Oracz, 2019).

Reactive oxygen species (ROS) have traditionally been viewed as destructive agents in plants; however, it has been recently explained that ROS also play a positive role in seed germination (Oracz and Karpiński, 2016). Oxygen can be transformed into multiple ROS, including singlet oxygen, superoxide, the hydroxyl radical, and hydrogen peroxide. Hydrogen peroxide is considered to be an important ROS agent, as it can pass readily through various cellular membranes (Anand et al., 2019). Reactive oxygen species production inside the seed transforms it from the quiescent seed produced by the mother plant into a biologically active seed that is capable of germination (Xia et al., 2018). This process occurs when environmental signals are accurately perceived and processed into endogenous signals by the seed (Bailly, 2019). The mitochondrion is known to carry out diverse functions in the cellular landscape. It synthesizes vitamins, (i.e., ascorbic acid, folic acid, and biotin) and selected amino acids (Foyer et al., 2020), and has a pivotal role in programmed cell death (PCD) (Zhao et al., 2018). Beside a major site for ROS production, mitochondrion also plays a significant role in seed germination (Bailly, 2019). Heat shock proteins localized in the mitochondria play a crucial role in seed germination through temperature-dependent ROS generation (Ma et al., 2019). This article mainly summarizes the roles of ROS, heat shock proteins and mitochondria in seed germination.

## Role of Reactive Oxygen Species in Seed Germination

Reactive oxygen species (ROS) are produced in the seed as a result of metabolism and play a significant role in seed germination (Table 1). Weakening of the endosperm is a prerequisite for the initiation of seed germination and is driven by various internal and external factors. The decay of the endosperm is directly linked to the production of ROS in response to the availability of external environmental signals (Zhang et al., 2019). ROS include free radicals such as singlet oxygen (^1^O^–2^), superoxide (O^–2^) or the hydroxyl radical (OH) (Bailly, 2019). Hydrogen peroxide is a reactive molecule that performs a signaling function in seed germination and has the ability to cross biological membranes (Hajihashemi et al., 2020). During the germination of the seed, the endogenous H_2_O_2_ accumulates inside the seed so the germination can occur, and seed coat can loosen up. When the production of H_2_O_2_ will begin to damage the internal organelles, the defense mechanisms inside the cell will be activated. Recently hydrogen peroxide responsive genes (*HRG*) 1 and 2 have been identified that play a significant role in containing the production of H_2_O_2_ inside the cell so that plant metabolic process cannot be disrupted (Gong et al., 2021). The specialty of *HRG1* and *HRG2* is that they will keep rather low content of protein until the level of H_2_O_2_ is elevated, which discerns them from the other hydrogen peroxide responsive genes providing novel pathway for H_2_O_2_ sensing and response. During the germination process, this novel pathway is involved in the meristem activity regulation of embryonic roots. The novel *HRG1* and *HRG2* genes express to protect the microenvironmental stability of root tip meristem for maintain meristem cell normal activity concerning both cell division and elongation (Gong et al., 2021). Abiotic and biotic stresses can result in oxidative stress that causes programmed cell death. In addition to their negative effects, ROS also play a significant positive role in dormancy release, seed germination signaling, protection against pathogens, and regulation of internal cellular machinery in response to external environmental dynamics. There is a specific “oxidative window” that allows cellular events to unfold in sequential order for seed germination if ROS are maintained within a particular range (Anand et al., 2019). To date, ROS dynamics have been studied mainly after water imbibition because they are easier to measure at this time.

**TABLE 1:** Effects of ROS on seed germination in various plant species.

Context	Effect	Species	References
Zn and arsenic stress	Negative	*Anadenanthera peregrina* and *Myracrodruon urundeuva*	Gomes et al., 2013
Germination	Positive	*Malus domestica*	Krasuska et al., 2014
Dormancy alleviation	Positive	*Malus domestica*	Dębska et al., 2013
Salt stress	Negative	*Arabidopsis thaliana*	Yang et al., 2019b
ABA cross talk	ABA positive regulator of rboh and ROS	*Arabidopsis thaliana*	Yang et al., 2019a
Cd stress	Negative	*Arabidopsis thaliana*	Nourimand and Todd, 2019
Mitochondrial functioning	Positive	*Arabidopsis thaliana*	Ma et al., 2019
Salt stress	Positive	*Arabidopsis thaliana*	Ortiz-Espín et al., 2017
Seed dormancy and iron deficiency	Positive	*Arabidopsis thaliana*	Murgia and Morandini, 2017
Germination/ABA	Negative	*Arabidopsis thaliana*	Baek et al., 2015
Salt stress/ethylene	Negative	*Arabidopsis thaliana*	Lin et al., 2013
Germination light	Positive	*Arabidopsis thaliana*	Lariguet et al., 2013
Dormancy	Positive	*Arabidopsis thaliana*	Leymarie et al., 2012
Germination/ABA	Positive	*Arabidopsis thaliana*	He et al., 2012
Germination/ABA/GA	Positive	*Arabidopsis thaliana*	Liu et al., 2010
Germination/ABA signaling	Positive	*Arabidopsis thaliana*	Bi et al., 2017
Dormancy/ABA/GA	Positive	*Hordeum vulgare*	Bahin et al., 2011
Seed germination and dormancy	Positive	*Hordeum vulgare*	Ma et al., 2017
Germination/ABA signaling	Positive	*Hordeum vulgare*	Ishibashi et al., 2017
Dormancy alleviation	Positive	*Hordeum vulgare*	Ma Z. et al., 2016
Germination/GA/NADPH oxidase	Positive	*Hordeum vulgare*	Kai et al., 2016
Germination/NADPH oxidase	Positive	*Hordeum vulgare*	Ishibashi et al., 2015
Dormancy	Positive	*Bidens Pilosa*	Whitaker et al., 2010
Dormancy alleviation	Positive	*Bunium persicum*	Amooaghaie and Ahmadi, 2017
Dormancy alleviation	Positive	*Hedysarum scoparium*	Su et al., 2016
Germination/endosperm weakening	Positive	*Lactuca sativa*	Zhang et al., 2014
Mutagen agents	Negative	*Zea Mays*	Zhang et al., 2018
Dormancy alleviation by heat	Positive	*Mesembryanthemum crystallimum*	Visscher et al., 2018
Drought and salt stress	negative	*Miscanthus*	Yang et al., 2018
Germination/ABA	Positive	*Pisum sativum*	Barba-Espin et al., 2012
Germination	Positive	*Pisum sativum*	Kranner et al., 2010
High temperature, drought stress	Negative	*Oryza Sativa*	Liu et al., 2019
Low phytic acid seed vigor	Positive	*Oryza Sativa*	Zhou et al., 2018
Dormancy alleviation (after ripening)	Positive	*Oryza Sativa*	Zhang et al., 2017
Germination NADPH oxidase	Positive	*Oryza Sativa*	Li et al., 2017
Osmotic and salt stress	Negative	*Oryza Sativa*	Chen et al., 2016
Germination/ABA/GA	Positive	*Oryza Sativa*	Ye and Zhang, 2012
Germination/ethylene	Positive	*Sorghum bicolor*	Ishibashi et al., 2013
Dormancy alleviation (after ripening)	Positive	*Helianthus annus*	Morscher et al., 2015
Dormancy alleviation/ABA/ethylene	Positive	*Helianthus annus*	El-Maarouf-Bouteau et al., 2015
Dormancy	Positive	*Helianthus annus*	Oracz et al., 2007
GA response	Positive	*Nicotina tabacum*	Oracz et al., 2009
Germination	Positive	*Vigna radiata*	Singh et al., 2015
Seed vigor and GA signaling	Positive	*Citrullus lanatus*	He et al., 2019
Dormancy	Positive	*Triticum aestivum*	Bykova et al., 2011

After ripening, seeds are quiescent and very low in moisture. Measurement of ROS production and metabolism in dry seeds is very challenging, mainly due to technical barriers. Oxygen is the main driver for ROS production in anhydrobiotic seeds. It typically exists in its ground state (^3^O_2_) with two unpaired electrons with parallel spins, and its reduction generates various forms of ROS. Oxygen is present primarily in the void spaces of dry seeds, and these dry spaces collectively form an air space network (Ahmed et al., 2018). Seeds are desiccated on the mother plant after ripening, but mother plants generate ROS to create an oxidative environment inside the seed. During seed storage, available oxygen kickstarts chemical reactions. Lipids can be easily oxidized in seeds under low moisture conditions and therefore serve as a source of free radicals. The increase in water uptake in zone 1 of the water sorption isotherm reduces lipid oxidation by filling the pore spaces and decreasing the oxygen concentration inside the seed, resulting in a low reaction rate. Lipid peroxidation in the low moisture system may lead to the reactions of proteins with lipid hydroperoxides, free radicals, and peroxide breakdown products (Cai et al., 2011). The key function of oxygen in the seed is the release of seed dormancy, as has been observed in experiments with barley, Arabidopsis, and sunflower (Leymarie et al., 2012; Beracochea et al., 2015).

The resumption of metabolic activity in the seed is related to the regulated enzymatic production of ROS. Seeds are very sensitive to water, which leads to the production of ROS. The decision to germinate is dependent on various environmental stimuli such as temperature, moisture, and light (Gomes and Garcia, 2013). Major sites involved in the production of ROS are the mitochondria, the peroxisome, and the NADPH oxidases of the plasma membrane. The inhibition of NADPH oxidase delays seed germination, underscoring the important function of this enzyme. After imbibition, the resurgence of mitochondrial respiration in the seed may cause electrons to be donated to oxygen as an electron acceptor, leading to ROS production (Kranner et al., 2010). NADPH oxidases, also known as respiratory burst oxidase homologs (Rbohs) after the homology with gp91^phox^ domain of the mammalian respiratory burst oxidase are main drivers of ROS production in the cell wall space which leads to seed germination through non-enzymatic way and results in radicle elongation and endosperm cap weakening in dicots (Sun L. R. et al., 2019). Rbohs transfers the electron from NADPH or NADH to apoplastic oxygen which results in the production of superoxide radicals and these radicals can directly cleave polysaccharides which will loosens the plant cell walls (Yang et al., 2020). As a typical dicot seed, i.e., *Lactuca sativa* has two layers of seed coverings, a dead and soft seed coat and an endosperm tissue covered with 2–3 layers of the living cells (Jones, 1974). NADPH oxidases as non-enzymatic mode along with enzymatic mechanisms, i.e., pectin-methyltransferases, cellulase and hemicellulose degrading enzymes are involved in the loosening and degradation of these layers so that the germination can occur (Zhang et al., 2014). Protein carbonylation and protein turnover increase owing to the accumulation of H_2_O_2_. It also causes a decrease in electron pressure in the mitochondrial electron transport chain, allowing the provision of reducing equivalents (NADPH) to the thioredoxin (Trx) system (via the pentose phosphate pathway), which is involved in the regulation of seed germination and seedling development (Li et al., 2017). The H_2_O_2_ concentration also affects the hormone balance by increasing GA and decreasing ABA/ethylene via 1-aminocyclopropane-1-carboxylic acid. This remodeling of hormone signaling may lead to the recommencement of metabolic activity that is essential for seed germination and seedling emergence (El-Maarouf-Bouteau and Bailly, 2008).

In monocots, the coleorhiza, a non-vascularized multicellular embryonic tissue, covers the seminal roots of monocot seeds and is thought to have a role in protecting the emerging root and it is also involved in the regulation of radicle emergence upon the germination of monocots seeds, i.e., wheat (González-Calle et al., 2015). The embryo is located on the side of endosperm, where it is considered that endosperm is involved in providing the nutrition to the growing embryo. During germination process, the epiblast and coleorhiza appears first and then the primary leaf appears from the coleoptile (Betekhtin et al., 2018). The role of coleorhiza is known to protect the emerging roots during seed germination; however, it has been established that it plays the same role in monocots which micropylar endosperm plays in the dicots, i.e., it acts as a barrier for radicle protrusion to complete germination (sensu stricto) (Holloway et al., 2021). The dissolution of coleorhiza occurs after 6h of imbibition of the water in rice and thus its degeneration is pre-requisite for the germination of the seeds (Barrero et al., 2009). The non-enzymatic mechanisms, i.e., the production of ROS is directly linked with the increase in the germination especially the accumulation and production of O^2–^, H_2_O_2_, and OH^–^ radicals were higher in coleorhiza and radicle than in the coleoptile of germinating seeds (Li et al., 2017). Moreover, NADPH oxidases (NOXs) are kind of proteins and are major enzymatic route of ROS synthesis in rice seed germination (Kai et al., 2016). The inhibition of NOXs in monocots can results in the delayed germination because these are involved in radicle and root growth. NOXs mRNAs are reported to express in the embryo and aleurone cells of barley seeds (Iglesias-Fernández et al., 2020), these expression sites are in conformity with the sites of ROS production in the seeds after imbibition (Ishibashi et al., 2015).

## Mitochondria Are Indispensable for Seed Germination

The mitochondrion is responsible for regulating various functions in the cell. It is involved in the production of vitamins (ascorbic acid, folic acid, and biotin) (Foyer et al., 2020), and selected amino acids so that cellular processes are carried out uninterrupted (Wagner et al., 2018). Moreover, under abiotic and biotic stress, it causes programmed cell death to put plant in a better position to combat these stresses. Mitochondria also induces the production of ROS which functions in the signaling pathways of cellular networks. It also has key roles in diverse metabolic pathways such as iron homeostasis, lipid metabolism, and nitrogen assimilation. Its most established role is in the production of cellular energy in the form of ATP through oxidative phosphorylation (Law et al., 2014). The onset of germination is marked by the conversion of simple, quiescent promitochondria of dry mature seeds into energetic, metabolically active, cristae-containing organelles. The study of sunflower seeds by transmission electron microscopy has shown that low density of the mitochondrial matrix, absence of a discontinuous outer membrane, and few cristae are associated with low ATP production through oxidative phosphorylation (Czarna et al., 2016). In other crops, such as rice (Taylor et al., 2010), maize (Bahaji et al., 2019), and peas (Henriet et al., 2021), the presence of mature mitochondria with large numbers of continuous cristae structures, an electron dense matrix, and abundant electron transport chain components were suggestive of high protein contents that enable the seed to produce large amounts of ATP through increased metabolic activity and respiration. Isolated promitochondria from rice seeds were rich in proteins but were unable to be metabolically active without imbibition. When rice seed was imbibed for 30 min, its mitochondrial metabolic and protein import functions were restored. Therefore, imbibition is a prerequisite for the transformation of promitochondria into mature and fully differentiated mitochondria. As seed germination proceeds, the import of components for mitochondrial function is reduced, and the import of machinery for primary metabolism in the cell increases (Best et al., 2020). The rate of protein import continues to rise, whereas the abundance of protein import machinery declines in the cell overall. This suggests that there may be some degradation of import machinery in the cell as the concentration of promitochondria decreases and that of mature mitochondria increases (Law et al., 2014).

## Changes in the Mitochondrial Transcriptome During Seed Germination

Dynamics of mitochondrial genes changes quantitatively as the germination progresses which validates that mitochondrial transcription and in turn the production of proteins is very vital for the germination of seeds. Northern blot analysis in maize showed that transcripts of genes associated with mitochondrial biogenesis such as *atpa*, *atp9*, *cox1*, *cox2*, and *cox3* were present at low abundance during early time points of seed germination but increased in abundance from 24 to 48 h after imbibition (HAI) (Logan et al., 2001). *Emp12* encodes a P-subfamily PPR protein that is located in the mitochondria (Small et al., 2004) and its expression is ubiquitous in a range of vegetative and reproductive tissues, which is mostly involved in the development of kernels (Cheng et al., 2016). The maize *Emp12* is involved in embryogenesis and endosperm development, the mutation in *Emp12* restricts the embryo and endosperm development causing embryo fatality. At the 16 DAP the kernels which were *Emp12* mutated were much smaller in size and microscopy study indicated that this mutation resulted in crumbled empty pericarp, accumulated less starch and the embryo development halted at the transitional stage, rendering an undifferentiated embryo (Sun F. et al., 2019). It suggests that mitochondrial localized *Emp12* plays an essential role in the embryogenesis and endosperm development. Moreover, the *Emp12* mutation causes trans-splicing of mitochondrial nad2 intron 2 and *cis*-splicing of nad2 intron4 are obstructed, whereas the *cis*-splicing of nad2 intron 1 is reduced in *Emp12* mutants (Cheng et al., 2016; Dai et al., 2018). It results in the dismantling of the complex1 assembly, and its activity decreases manifolds in the mutants and the expression of other alternative oxidases, and several other mitochondrial complexes greatly increases (Xiu et al., 2016; Qi et al., 2017). Therefore, *Emp12* is inevitable for the accurate and timely trans-splicing of nad2 intron 2 and cis splicing of nad2 introns 1 and 4 and plays a significant role in the complex 1 biogenesis, embryogenesis and endosperm development in maize (Sun F. et al., 2019).

**FIGURE 2:**
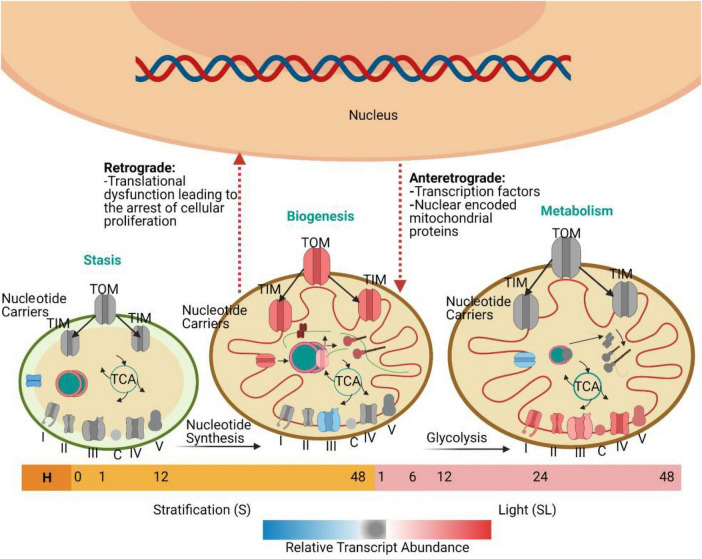
Mitochondrial biogenesis during seed germination, based on the work of Law et al. (2012). Seeds were collected at ten time points: H (freshly harvested); 0 h (dry seeds after two weeks of desiccation); 1 h S, 12 h S, and 48 h S [three time points during seed stratification (S)]; and 1 h SL, 6 h SL, 12 h SL, 24 h SL, and 48 h SL [five time points during the exposure to continuous light after stratification (SL)]. The promitochondria in dry seeds do not have the cristae that are associated with mature mitochondria. The transient expression of transcripts encoding proteins associated with DNA and RNA metabolism and nucleotide synthesis and import occurs when the seed is shifted from stratification to continuous light. Afterward, there is transient expression of transcripts encoding proteins for protein metabolism and import functions. During the next stage, after 24 h of continuous light, there is increased expression of genes encoding various metabolic components associated with the Tricarboxylic acid (TCA) cycle and the electron transport chain. The progress of each minute event in this process is closely monitored by the nucleus and mitochondrion through processes known as antegrade and retrograde regulation. TIM, the inner membrane; TOM, the outer membrane.

Similarly, a study in rice demonstrated that there was a low concentration of mitochondrial transcripts 3 HAI and a peak in transcripts related to mitochondrial energy production and metabolism at 12 HAI (Howell et al., 2009). Transcriptome data indicated that a small surge in transcripts in the germinating seed at 3 HAI was specifically associated with mitochondrial proteins rather than any other organelle, suggesting that the activation of mitochondria is essential for seed germination after imbibition (Logan et al., 2001). The analysis of genes that encode mitochondrial proteins has demonstrated a triphasic progression of transcriptomic events during seed germination (Law et al., 2012). Initially, two groups of genes are transiently expressed. The first group shows the highest transcript abundance until the end of stratification and before the seed is exposed to continuous light. They encode proteins that function in nucleic acid metabolism. The second group encodes proteins with import and synthesis functions. They are followed by a third group of genes that encode electron transport chain components and whose transcript abundance progressively increases. During the first transcriptomic phase, the first two groups of genes are activated, and the abundance of proteins related to DNA and RNA metabolism increases. These include proteins that function in transcription, RNA editing, splicing, processing, stability, and translation. Specifically, there is over representation of mitochondrial targeted pentatricopeptide repeat proteins (PPRs). PPRs are mitochondria or plastid-targeted proteins with diverse functions in RNA metabolism: RNA editing (Chateigner-Boutin et al., 2008), transcription (Ding et al., 2006), splicing (De Longevialle et al., 2008), processing (Nakamura et al., 2003), stability (Yamazaki et al., 2004), and translation (Choquet and Wollman, 2002). The expression of these transcripts is synchronized with the expression of transcripts encoding proteins that function in cytosolic nucleotide metabolism and factors responsible for conveying these nucleotides into the mitochondrial matrix. This pattern of transcription suggests that the coordinated production and transport of nucleotides is important, enabling them to be used for the transcription of mitochondrial genes (Li et al., 2012). During the second phase, the second group of genes is activated, including transcripts that encode ribosomes, translation factors, and tRNA-related functions (Narsai et al., 2011). There is an increased abundance of proteins related to mitochondrial protein import, including components of the inner membrane (TIM) and the outer membrane (TOM) complexes. This suggests that the import of nuclear-encoded mitochondrial proteins goes hand-in-hand with the synthesis of organelle-encoded proteins. This is necessary because many mitochondrial protein complexes contain both nuclear- and organelle-encoded subunits, and their successful assembly requires careful control of subunit accumulation (Jänsch et al., 1996). The presence of these biogenesis factors early in the germination time course highlights the significance of mitochondrial biogenesis for successful seed germination (Law et al., 2012). The third group of mitochondrial transcripts encodes components of the electron transport chain. The abundance of these proteins increases as germination progresses and it peaks before the end of germination. This marks the third and final phase in the transition of promitochondria to mature mitochondria and establishes the metabolic functions that are required for the vegetative stages of plant development (Figure 2) (Howell et al., 2009).

## Changes in the Mitochondrial Proteome During Seed Germination

Numerous mitochondrial proteins are involved in seed germination; they perform various functions such as respiration, metabolism, import or transport, carbon metabolism/-tricarboxylic acid cycle (TCA) reactions, stress responses, and chaperone activities. The abundance of these proteins in seed mitochondria at the time of germination indicates that they are indispensable for seed germination (Hao et al., 2019). Galland performed a proteome study in the mitochondria of Arabidopsis seeds within the first 0–24 h of germination (Galland et al., 2014). Two hundred fifty-seven non-redundant proteins from 475 identified mitochondrial protein spots showed various responses during seed germination. There were mitochondrial proteins that were up- or down regulated or remained at a constant level during the seed germination. The mitochondrial proteins which were upregulated were glutamate dehydrogenase 1 or 3, monodehydroascorbate reductase, glyceraldehyde-3-phosphate dehydrogenase, succinate-semialdehyde dehydrogenase, the beta subunit of ATP synthase, aconitate hydratase 3, phosphoenolpyruvate carboxykinase, the beta subunit of mitochondrial processing peptidase, Hsp 60, and translation elongation EF-Tu. The proteins which remained at constant levels from 0–24 h, including succinyl-CoA ligase alpha-chain, the flavoprotein subunit of succinate dehydrogenase, and Hsp 70-2. A few newly synthesized proteins decreased in abundance over the 24-h germination period, such as the alpha subunit of ATP synthase and superoxide dismutase 2 (Czarna et al., 2016). Some proteins that were not newly synthesized but decreased over the germination period included the NADH-ubiquinone oxidoreductase 75 kDa subunit, superoxide dismutase 1, and late embryogenesis abundant proteins. The relative abundance of specific proteins at different time points and selective mRNA translation highlight those proteins that are important for the process of seed germination (Rao et al., 2017).

## Heat Shock Proteins Are the Ultimate Decision-Makers in Seed Germination

In addition to their established role in protection from heat, heat shock proteins play a significant part in seed germination and development (Kaur et al., 2016). Seed germination depends on the surrounding temperature, which can delay or expedite the process after sowing (Ding et al., 2020). The germination efficiency of the cotton seed is related to warm temperatures in a narrow window ranging from 20°C to 36°C (Zhang et al., 2015; Ma W. et al., 2016). The functional characterization of *NnHSP17.5* gene in sacred lotus (Nelumbo nucifera Gaertn) has revealed that heat shock proteins (HSPs) play an important role in the seed germination and in the protection of the seeds against heat stress (Zhou et al., 2012). NnHSP17.5 is a member of class 2 proteins which are localized in cytoplasm and nucleoplasm, and specifically expressed in seeds development at later stages under normal conditions and are strongly up regulated in the germinating seeds upon heat and oxidative stresses (Scharf et al., 2001). When the heat stress goes beyond 42°C, the NnHSP17.5 expression gets instantly upregulated and in case if there is high concentration of H_2_O_2_ causing the oxidative stresses then again the NnHSP17.5 expressions in the germinating seed increases (Bailly et al., 2008). The role of small HSP17.4 as a regulator of thermotolerance during the seed development has shown in *Arabidopsis thaliana* seeds that the seeds which indicated high tolerance against desiccation had high concentration of sHSP17.4 during seed development in comparison to the mutants which were producing low concentration of sHSP17.4. Moreover, the study indicated that the seeds indicated low dormancy when the concentration of HSP17.4 was high or equal to the wild type in comparison to the mutant which expressed reduced concentration of HSP 17.4 (1%–2% of wild type) or at undetectable levels (Wehmeyer and Vierling, 2000).

Mitochondria I subfamily small heat shock proteins (msHSPs) are significantly induced during seed germination, especially GhHSP24.7 in cotton. There was a positive correlation (*R* = 0.99) between GhHSP24.7 expression levels and the change in temperature from 4°C to 36°C, underscoring the importance of this HSP in seed germination. Likewise, *GhHSP24.7* overexpression lines showed faster germination than those in which *GhHSP24* expression was suppressed (Ma et al., 2019). This result confirmed that HSPs have a significant role in seed germination (di Donato and Geisler, 2019). Heat shock proteins regulate seed germination in response to temperature dynamics. For example, GhHSP24.7 accelerates seed germination by 50% when the temperature is increased from 20°C to 36°C (Ma et al., 2019). However, the germination of various crop plants begins to decline if temperatures increase above an optimum range. The germination percentage of wheat decreased when the temperature rose above 30°C, and its germination percentage at 45°C was only 12% (Essemine et al., 2010).

Heat shock proteins (HSPs) also play a key role in the weakening of the testa so that the embryo can achieve its growth potential (Wehmeyer and Vierling, 2000). Seeds from GhHSP24.7 suppression lines had higher puncture force of endosperm (PFE) values than seeds with normal levels of GhHSP24.7. High PFE indicates a strong penetration resistance of the seed covering layers, and seeds in which GhHSP24.7 was suppressed showed both high PFE and delayed germination. ROS is a dominant factor in the transformation of the quiescent seed to a metabolically active organism, and GhHSP24.7 improves seed germination by increasing ROS production. Ma et al., observed that seeds with high levels of GhHSP24.7 expression underwent timely germination, whereas seeds with low GhHSP24.7 expression produced less H_2_O_2_ and O^2–^, which delayed germination (Ma et al., 2019). GhHSP24.7 also influenced the cellular structure of the seeds. Its high expression in seeds indicated that the two layers of cells in the endosperm were beginning to separate, marking the decay of cell structure in the micropylar endosperm. The function of HSPs is conserved in various plants such as tomato and Arabidopsis, as demonstrated by the presence of functional orthologs in the two genomes. Two Arabidopsis mitochondrial sHSPs (AtHSP23.5 and AtHSP23.6) and two tomato mitochondrial sHSPs (SlHSP23.8B and SlHSP21.5B) are reported to have definite roles in seed germination (Ma et al., 2019).

In summary, seed dormancy and seed germination respond to an interplay of endogenous and exogenous factors (Liu and Hou, 2018).

## Conclusion

Uniform seed germination is essential for maximizing crop production. Seed germination is a very complex process that requires the careful regulation of external and internal biotic and abiotic interactions. The accurate and optimized coordination of phytohormones, light, temperature, heat shock proteins, ROS, and endosperm decay is required to permit testa rupture and subsequent germination. Seed germination therefore requires internal responsiveness to external environmental cues.

## Author Contributions

MF, XZ, and MZ wrote the manuscript. WM and JZ revised the manuscript. All authors contributed to the article and approved the submitted version.

## Conflict of Interest

The authors declare that the research was conducted in the absence of any commercial or financial relationships that could be construed as a potential conflict of interest.

## Publisher’s Note

All claims expressed in this article are solely those of the authors and do not necessarily represent those of their affiliated organizations, or those of the publisher, the editors and the reviewers. Any product that may be evaluated in this article, or claim that may be made by its manufacturer, is not guaranteed or endorsed by the publisher.

## Abbreviations

ABA: Abscisic acid
GA: Gibberellins
PCD: Programmed cell death
ROS: Reactive oxygen species
ET: Ethylene
DOG: Delay of Germination
QTL: Quantitative trait loci
ME: Micropylar endosperm
TCA: Tricarboxylic acid cycle
HAI: Hours after imbibition
PPRs: Pentatricopeptide repeat proteins
HSP: Heat shock protein
PFE: Puncture force of endosperm
NOX: NADPH oxidase.
